# Effect of insertion layer on electrode properties in magnetic tunnel junctions with a zero-moment half-metal

**DOI:** 10.1038/s41598-019-40609-3

**Published:** 2019-03-11

**Authors:** Aleksandra Titova, Ciarán Fowley, Eugene Clifford, Yong-Chang Lau, Kiril Borisov, Davide Betto, Gwenael Atcheson, René Hübner, Chi Xu, Plamen Stamenov, Michael Coey, Karsten Rode, Jürgen Lindner, Jürgen Fassbender, Alina Maria Deac

**Affiliations:** 1 Institute of Ion Beam Physics and Materials Research, Helmholtz - Zentrum Dresden - Rossendorf, Dresden, Germany; 2 Institute for Physics of Solids, Technische Universität Dresden, Dresden, Germany; 3 CRANN and School of Physics, Trinity College Dublin, Dublin 2, Ireland

## Abstract

Due to its negligible spontaneous magnetization, high spin polarization and giant perpendicular magnetic anisotropy, Mn_2_Ru_*x*_Ga (MRG) is an ideal candidate as an oscillating layer in THz spin-transfer-torque nano-oscillators. Here, the effect of ultrathin Al and Ta diffusion barriers between MRG and MgO in perpendicular magnetic tunnel junctions is investigated and compared to devices with a bare MRG/MgO interface. Both the compensation temperature, *T*_comp_, of the electrode and the tunneling magnetoresistance (TMR) of the device are highly sensitive to the choice and thickness of the insertion layer used. High-resolution transmission electron microscopy, as well as analysis of the TMR, its bias dependence, and the resistance-area product allow us to compare the devices from a structural and electrical point of view. Al insertion leads to the formation of thicker effective barriers and gives the highest TMR, at the cost of a reduced *T*_comp_. Ta is the superior diffusion barrier which retains *T*_comp_, however, it also leads to a much lower TMR on account of the short spin diffusion length which reduces the tunneling spin polarization. The study shows that fine engineering of the Mn_2_Ru_*x*_Ga/barrier interface to improve the TMR amplitude is feasible.

## Introduction

**Figure 1:**
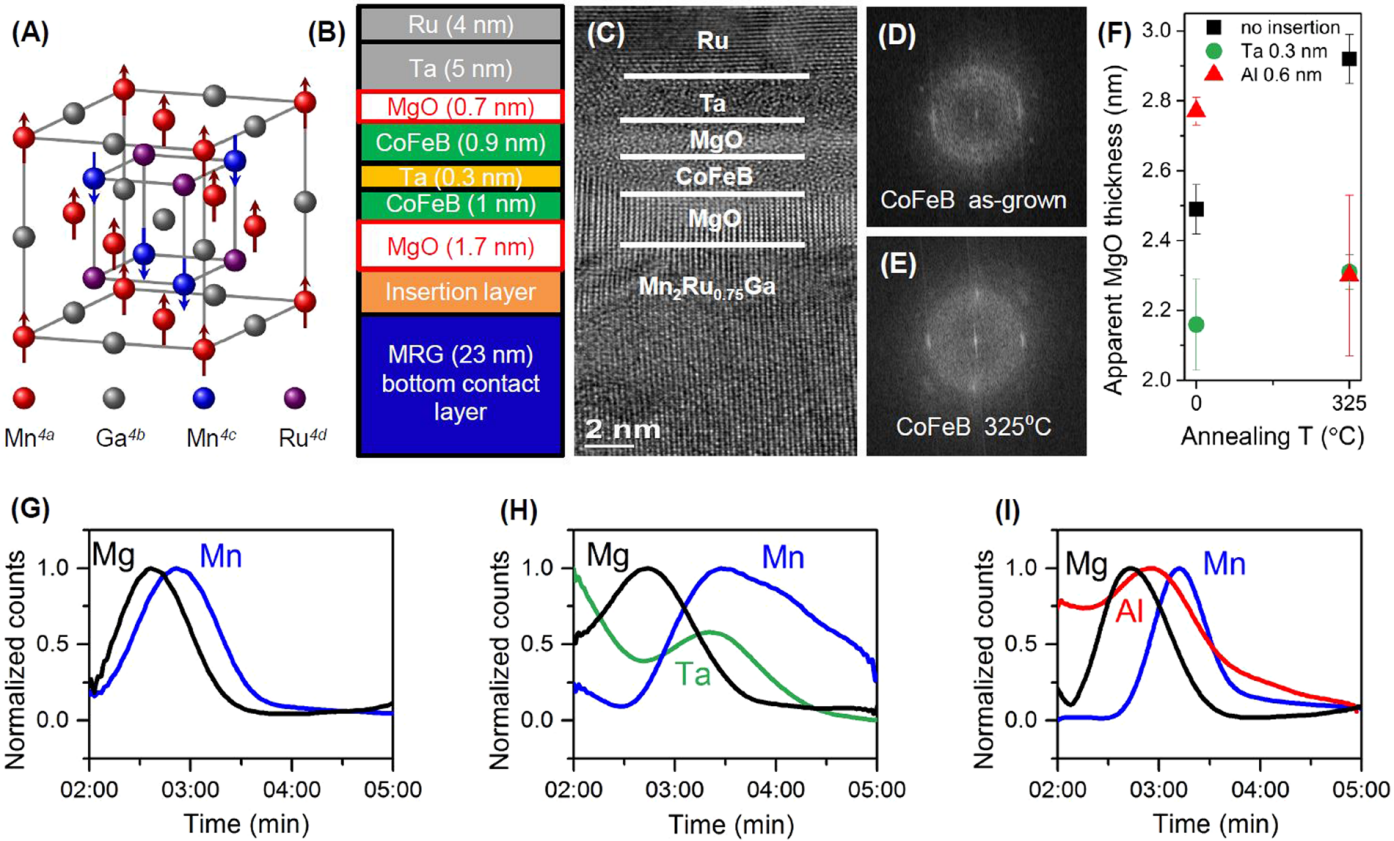
(**A**) Crystal structure of MRG. (**B**) Schematic of fully perpendicular MTJ multilayer stack structure. (**C**) High-resolution TEM image of the whole MTJ structure. Fast Fourier Transform of the CoFeB layer in the as-grown state (**D**) and after annealing at 325 °C (**E**). (**F**) Apparent MgO thickness dependence for the samples with different insertion layers. SIMS spectra recorded during device fabrication; initial Mn peaks (blue line) show that Mn is present in the MgO barrier for devices with the no diffusion barrier (**G**) or with Al diffusion barrier (**I**), as opposed to the case when Ta is used (**H**).

In order to respond to the social need to transmit ever larger amounts of data and increase data transmission speed, cheap and compact THz-transmitters/receivers have to be created^1–3^. Spin-transfer-torque nano-oscillators (STNOs) can be a good solution for this demand^4,5^. In such devices, a spin-polarized current induces dynamics of the magnetization^6,7^. The operating frequencies for current STNOs based on typical transition metal-based ferromagnets and their derivatives, lie in the GHz range^8^, due to the low magnetic anisotropy and high magnetization. Materials with ultra-high effective anisotropy fields are very promising, as they exhibit magnetic resonances of several hundred GHz^9–11^. By integrating such materials into STNOs, sub-THz emission may be achieved. In order to obtain high output power, the multilayer stacks have to exhibit high magnetoresistive effects: giant magnetoresistance (GMR)^12,13^ or tunneling magnetoresistance (TMR)^14,15^. High spin polarization is a necessary condition for strong magnetoresistive effects^16–18^, and therefore half-metals, which are metallic for one spin direction and semiconducting for the other, appear as the ideal choice^19^. Additionally, the perfect electrode material should exhibit as little stray field as possible and be largely insensitive to external magnetic fields^20–22^. These requirements can be satisfied if the saturation magnezation (*M*_s_) of the material is close to zero, since namely *M*_s_ is in the origin of the unwanted shape-dependence of the magnetic properties, and *M*_s_ is responsible for energy losses during the switching. A zero-moment magnet produces no stray field and is free of shape anisotropy, thereby effectively removing the obstacles mentioned above. Here, we focus on high anisotropy compensated ferrimagnetic half-metals (CFHMs) – a class of materials predicted in 1995 by van Leuken and de Groot^23^. CFHMs behave like antiferromagnets (AFMs) with respect to external magnetic fields, since the magnetic moments of the two sublattices compensate, while simultaneously exhibiting half-metallic electron transport behavior. Many attempts had been made to fabricate such materials, and although half-metallicity^24^ and high magnetic anisotropy^25^ were observed for Co_2_-based Heusler compounds, the magnetic moment per unit cell was high^26^. Currently, MnGa-based alloys, where high anisotropy is coupled with low magnetization, attract intense attention^27–29^ and some have already been integrated into magnetic tunnel junctions (MTJs)^30–32^. Complete magnetic compensation in such alloys is, however, difficult to obtain. MnPtGa was shown to exhibit zero net moment, but it does not show half-metallicity, possibly due to the tetragonal distortion of the crystal unit cell^33^. In 2014, Mn_2_Ru_*x*_Ga (MRG) was shown to combine the ideal properties of low magnetization, high magnetic anisotropy and large spin polarisation^34^. It crystallises in a near-cubic Heusler structure with two antiferromagnetically coupled manganese sublattices, located on the 4*a* and 4*c* Wyckoff positions, respectively (see Fig. 1(A)). We will refer to the Mn sublattices as Mn_4*a*_ and Mn_4*c*_. Ga occupies 4*b* positions and the dopant Ru occupies a fraction of the remaining free 4*d* positions. Ru doping and temperature do not appreciably affect the properties of the Mn_4*a*_ sublattice, only that of Mn_4*c*_^35^. Due to the crystallographic differences between the two magnetic sublattices, complete magnetic compensation and, thus, strictly zero moment is possible and occurs at a precise temperature, *T*_comp_, which depends on both Ru concentration and bi-axial substrate-induced strain^36^. MRG has also been successfully integrated into perpendicular MgO-based MTJs, where low-bias TMR reaching up to 40% at 10 K^37^ was achieved when a thin 0.6 nm Al layer was inserted between MRG and the MgO barrier. While temperature-dependent TMR^37^ and anomalous Hall effect (AHE)^38^ show that electronic transport is governed by the Mn_4*c*_ sublattice only, magnetometry measurements are needed to accurately yield *T*_comp_. Furthermore, since *T*_comp_ is governed by the magnitudes of both the Mn_4*a*_ and Mn_4*c*_ sublattices, it is extremely sensitive to the changes in the sublattice moments with respect to material diffusion and its analysis in unpatterned multilayer stacks is also required.

It is first prudent to introduce how the changes in *T*_comp_ can be used to deduce changes in sublattice properties. It was shown by DFT calculations that considerable site disorder can be observed in MRG, particularly on the Mn_4*a*_ site leading to a Mn deficiency, which will increase *T*_comp_. In ref.^39^, half-metallicity is proposed with a Mn/Ga ratio of 1.4 as a result of a larger number of Mn/Ga anti-sites, and a Ru concentration of 0.7. Assuming the Mn is lost solely from the Mn_4*a*_ site through disorder the observation will be a higher value of *T*_comp_^40^. The loss of Mn on the 4*c* sublattice, on the other hand, will lead to a decrease in *T*_comp_ from the expected value.

In ref.^37^ MRG was already integrated into an MTJ device, but in that paper only the results with an Al 0.6 nm insertion layer between the CFHM and MgO were presented. Here, we expand the investigation to different diffusion barrier materials and also investigate in detail the magnetic properties of the CFHM in complex multilayers. We show that the addition of Ta between MRG and MgO gives the best electrode properties but reduces the tunneling spin polarization due to its low spin diffusion length. Al on the other hand, gives rise to the largest TMR and the thickest effective tunnel barrier indicating that either a hybrid AlOx/MgO or a MgAl_2_O_4_ barrier is formed^41,42^. Without a diffusion barrier, the effective barrier thickness is also increased, suggesting interfacial MRG oxidation, and a low TMR is observed in these stacks.

## Results and Discussion

### Multilayer growth and structural characterization

Different Mn_2_Ru_*x*_Ga compositions with *x* = 0.65, 0.75, 0.9, and 1.1 were grown by varying the sputtering power on a Mn_2_Ga target while keeping that of Ru constant. All depositions were carried out in a “Shamrock” cluster deposition tool with a base pressure of less than 1 × 10^−7^ mbar. Changing the Ru concentration in MRG allows adjusting the *T*_comp_ from 2 to 450 K^34^. Due to the biaxial strain induced during growth^36^, MRG possesses an out-of-plane easy axis magnetization along the crystallographic *c*-direction. Following the deposition of 23 nm of MRG, different diffusion barriers with varying thicknesses (0.3 nm, 0.6 nm, 0.9 nm), were grown followed by an MgO tunnel barrier with a design thickness of 1.7 nm. We have chosen Ta and Al as insertion layers, as they oxidize rapidly and form stable oxides, which are expected to prevent the oxidation of MRG, as well as diffusion of Mn into the tunnel barrier. We also fabricated devices without insertion layers. Finally, a composite top electrode of CoFeB/Ta/CoFeB/MgO/Ta/Ru was deposited. This structure was chosen, as it provides two MgO/CoFeB interfaces with strong perpendicular magnetic anisotropy (PMA), it is stable against surface roughness and exhibits high thermal stability^43^. The complete stack is illustrated in Fig. 1(B). Cross-sectional transmission electron microscopy (TEM) was used to study the crystalline structure of the multilayer stack, see Fig. 1(C). MRG, as well as the MgO tunnel barrier, show a strong (001) texture, whereas CoFeB is only partly crystalline in the as-grown state (Fig. 1(D)). Subsequent vacuum annealing at 325 °C for 1 hour leads to enhancement of CoFeB crystallinity (Fig. 1(E)), and improved PMA^44^. None of the sub-nm diffusion barriers were clearly visible in the images, but their effect can be seen as an (apparent) increase of the MgO thickness (Fig. 1(F)). The thickest composite MgO barrier was observed with the Al 0.6 nm. The thinnest barrier is observed with Ta 0.3 nm, close to the nominal MgO thickness. This observation indicates that, structurally, the Ta barrier prevents material diffusion from MRG to MgO. Indeed, secondary ion mass spectrometry (SIMS), recorded during microfabrication of devices, revealed that Mn was present in the MgO for the samples with no and with Al insertion, while this was not the case for the films with Ta insertion, Fig. 1(G–I). Besides, it is useful to analyze the elements in terms of their standard oxidation potential (SOP) *E*°. According to the values of SOP, the metals are ranked from highest to lowest as; Al with *E*° = 1.662 V, leading to the possible formation of either a hybrid AlOx/MgO or a MgAl_2_O_4_ barrier^41,42^; Mn with *E*° = 1.185 V, meaning that the bare MRG/MgO interface will also oxidize, and; finally, Ta with *E*° = 0.6 V, implying that compared to the other two, we can assume that Ta stays metallic.

### Magnetic properties

**Figure 2:**
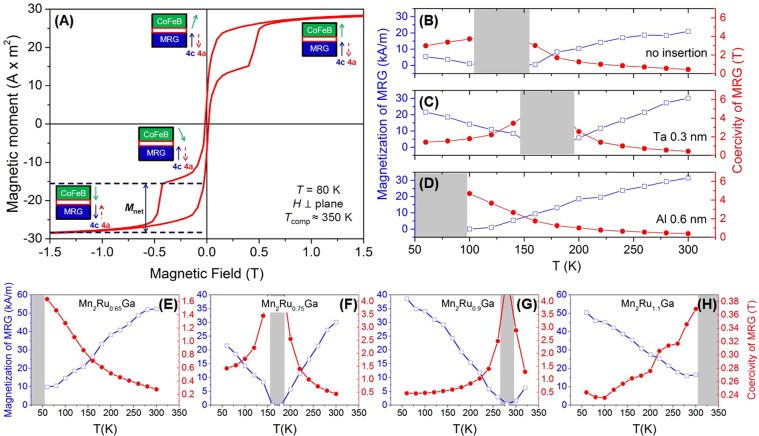
(**A**) Out-of-plane hysteresis loop of Mn_2_Ru_0.9_Ga/MgO/CoFeB MTJ at 80 K with Ta 0.3 nm insertion layer. (**B**–**D**) Temperature dependence of the net magnetization (blue) and the coercive field (red) for Mn_2_Ru_0.75_Ga-based MTJs without any diffusion barrier (**B**), with Ta 0.3 nm (**C**) and Al 0.6 nm (**D**) insertion layer. (**E**–**H**): Determination of *T*_comp_ for the MTJs with Ta 0.3 nm insertion layer and different Mn_2_Ru_*x*_Ga composition (*x* = 0.65 (**E**); 0.75 (**F**); 0.9 (**G**); 1.1 (**H**)).

Superconducting quantum interference device (SQUID) magnetometry was used to determine the effect of the diffusion barriers on the coercivity, µ_0_*H*_c_, and net magnetic moment of MRG, *M*_net_, in the range 60 K < *T* < 300 K. A typical magnetization versus applied magnetic field (M-H) curve for a multilayer with *x* = 0.9 and no diffusion barrier, recorded at 80 K, is shown in Fig. 2(A). The individual sublattice magnetization directions of MRG are depicted in the figure. Below *T*_comp_, as it is the case at 80 K, the Mn_4*c*_ sublattice moment (blue arrow) is parallel to *M*_net_^38^, while the Mn_4*a*_ moment (red arrow) is oriented antiparallel. As the magnetic field is swept from +7T to −7T, the magnetic moment of CoFeB (green arrow) rotates around zero field. The sharp jump observed at −0.4T corresponds to the µ_0_*H*_c_ of MRG (Mn_4*c*_ and Mn_4*a*_ reverse their directions simultaneously, and maintain their antiparallel alignment). The change in magnetic moment therefore corresponds to the *M*_net_ of MRG (vertical black arrow). Figure 2(B–D) plot the evolution of µ_0_*H*_c_ (red circles) and magnetization (blue open squares) for multilayers without an insertion layer (B), a Ta 0.3 nm (C) and an Al 0.6 nm (D) insertion layer, respectively, over the entire temperature range. The bottom electrode composition for all three samples is the same, Mn_2_Ru_0.75_Ga. At *T*_comp_, *M*_net_ goes to zero and µ_0_*H*_c_ diverges^45^; it lies in the grey shaded region in the graphs. The Ta 0.3 nm diffusion barrier (Fig. 2(C)) results in the highest *T*_comp_.

For the Ta 0.3 nm diffusion barrier, µ_0_*H*_c_ and *M*_net_ are plotted in Fig. 2(E–H) versus temperature for different values of *x*. Consistent with the previous work^36^, a lower Ru composition results in a lower *T*_comp_. For all values of *x* the shift of *T*_comp_ from the expected value is about 60 K. The slightly lower *T*_comp_ is attributed to the modification of the number of Mn_4*c*_ anti-sites, thereby reducing *T*_comp_^39^. MRG’s *T*_comp_ can be recovered by post-annealing^40^ and a 20 K rise is observed after annealing at 325 °C for 1 hour. Most likely, annealing results in a loss of Mn atoms from the 4*a* sublattice resulting in a higher compensation temperature for MRG.

### Electronic transport properties

**Figure 3:**
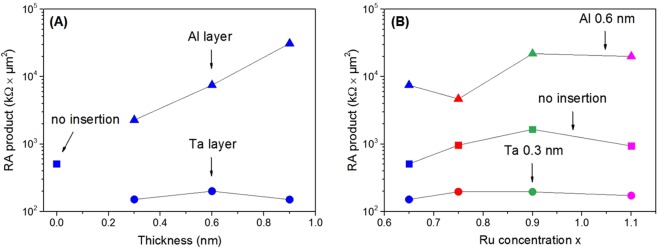
RA product of 20 × 20 μm^2^ MRG-based MTJs (**A**) with *x* = 0.65 as a function of insertion layer thickness, (**B**) as a function of Ru concentration. The measurements were done with 200 mV bias applied.

The multilayers were patterned into 6 × 6 µm² and 20 × 20 µm² MTJs to measure magnetotransport as function of applied magnetic field and bias voltage. Measurements were carried out at room temperature using a dc two-probe method with the magnetic field applied perpendicular to the sample surface. TMR was only observed for the devices without an insertion layer, and for MTJs with Ta 0.3 nm and Al 0.6 nm insertions. For the other stacks, only AMR attributed to CoFeB was detected. Transport measurements show that insertion of any diffusion barrier leads to significant changes in the resistance-area (RA) product of the MTJs (Fig. 3). The use of Al leads to a higher RA product than the samples without diffusion barrier. The RA product increases with Al thickness (Fig. 3(A)) indicating that the effective thickness of the barrier is increasing, similar to what was observed in TEM analysis. On the contrary, Ta results in a lower RA product and is independent of the diffusion barrier thickness. This is in good agreement with our structural studies, where the thinnest tunnel barrier was observed with Ta 0.3 nm insertion. Since the RA is low and the tunnel barrier thickness is close to the expected after the deposition value we conclude that Ta is preventing Mn diffusion into the MgO and interfacial MRG oxidation. The Ru concentration in the electrode does not significantly affect the RA product (Fig. 3(B)).

**Figure 4:**
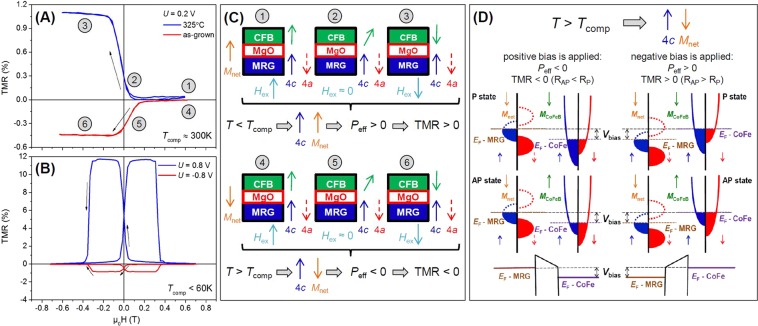
The TMR curves measured while applying magnetic field perpendicular to the film plane at room temperature for (**A**) Mn_2_Ru_0.9_Ga/MgO/CoFeB sample and (**B**) Mn_2_Ru_0.75_Ga/Al(0.6nm)/MgO/CoFeB sample. (**C**) Schematic diagram of the magnetic moment state changing with applied field. (**D**) Representation of the spin split density of states of MRG and CoFeB for different polarity of applied voltages and different relative orientation of the two magnetic layers corresponding to the panel (B). The MRG spin ↑ pocket is attributed to the Mn_4*c*_ sites, and the spin ↓ pocket is attributed to the Mn_4a_ sites.

Examples of measured TMR curves are shown in Fig. 4. We define TMR as the resistance difference between antiparallel (R_AP_) and parallel (R_P_) relative orientations of MRG’s and CoFeB’s magnetic moments, normalized by R_P_: (R_AP_ − R_P_)/R_P_ × 100%. First, we examine the effect of annealing on the sample with Mn_2_Ru_0.9_Ga bottom electrode and no insertion layer (Fig. 4(A)). The applied bias voltage is +0.2 V. SQUID measurements on this sample showed that the compensation temperature for this MRG composition is close to room temperature. The coercivity of MRG at room temperature is above 600 mT, therefore only the CoFeB moment reversal around zero field is seen for the entire measurement range. In the as-deposited state (red line), the resistance is higher for parallel alignment (positive applied magnetic field) and, thus, the TMR is negative. Upon annealing (blue line), the opposite response is seen and the resistance is found to be higher for negative applied magnetic field (when antiparallel state is reached), leading to the positive TMR. The change in sign of TMR is the result of a shift in *T*_comp_ from slightly below room temperature to slightly above, as a result of the annealing process. We also examined the effect of the applied bias for a sample with Mn_2_Ru_0.75_Ga (Fig. 4(B)) that has been annealed at 325 °C. In this sample, the *T*_comp_ is far below room temperature, therefore 300 mT is already enough to reverse *M*_net_ of MRG, promoting parallel alignment of the magnetic moments of CoFeB and MRG. When the applied voltage is negative (−0.8 V, blue line), corresponding to the tunneling of electrons from CoFeB into MRG, the TMR is 12% and positive. If +0.8 V is applied (red line), electrons are tunneling from MRG to CoFeB, the TMR is negative and possesses a smaller value compared to the other bias voltage polarity. The TMR values are much lower than is reported for Fe/MgO/Fe-like junctions, and this is attributed to the quality of the tunnel barrier rather than reduced spin polarization of the electrode. MRG has already been shown to be highly spin polarized^34^.

The changes of the TMR sign in Fig. 4(A,B) can be attributed to a sign change of tunnelling spin polarization (TSP). Since the spin polarization sign of CoFeB is always positive, following Julliere’s model^16^, the TMR is positive or negative when the TSP of MRG electrode is positive or negative, respectively. The TSP’s change of the sign can be explained using a simple model in which the tunnelling current is the sum of independent spin-polarized tunnelling currents from the Mn_4*a*_ and Mn_4*c*_ sublattices. Indeed, scanning tunnelling microscopy studies showed that the tunnelling current can significantly vary between neighboring atomic sites in metallic alloys^46^. The sign of MRG spin polarisation depends on the orientation of the respective sublattice magnetization with respect to the *M*_net_^47^. The response of all devices with regards to *T*_comp_ being above or below room temperature is explained using the model in Fig. 4(C). The magnetization states are numbered 1 through 6 in panel (C) and are indicated on the TMR curves in panel (A).

The magnetization of MRG is separated into its constituent sublattices, Mn_4*a*_ and Mn_4*c*_, depicted as blue and red arrows respectively. Below *T*_comp_, the Mn_4*c*_ sublattice is in the same direction as the *M*_net_ (see the upper panel in (C)). Since only Mn_4*c*_ contributes to the magnetotransport, then the TSP will be positive only when Mn_4*c*_ and *M*_net_ are parallel resulting in positive TMR. Above compensation, the situation is reversed (lower panel in (C)) and the TMR response will be negative due to the antiparallel alignment of Mn_4c_ and *M*_net_. However, since transport measurements only involve states at or in close vicinity to the Fermi level, by altering the measurement voltage we are able to scan around *E*_F_ and thus alter the effective tunnelling spin polarisation of MRG, *P*_eff_, and change the TMR response from positive to negative^37^. Particularly in Fig. 4(B), where MRG exhibits a *T*_comp_ below room temperature, *M*_net_ follows the magnetic moment of the Mn_4*a*_ sublattice, high positive voltage shifts the accessible states in MRG towards the Fermi level of CoFeB and the reversal of *P*_eff_ from positive to negative is observed due to voltage-driven access to the Mn_4*a*_ pocket as illustrated in Fig. 4(D). In the case of positive voltage applied, the conductance of the parallel (P state) is lower than the conductance in the antiparallel state (AP state). The opposite situation was observed for negative voltages, thereby leading to the positive TMR sign. We can track the TMR response for different applied voltages to observe the direction of *P*_eff_ in detail.

**Figure 5:**
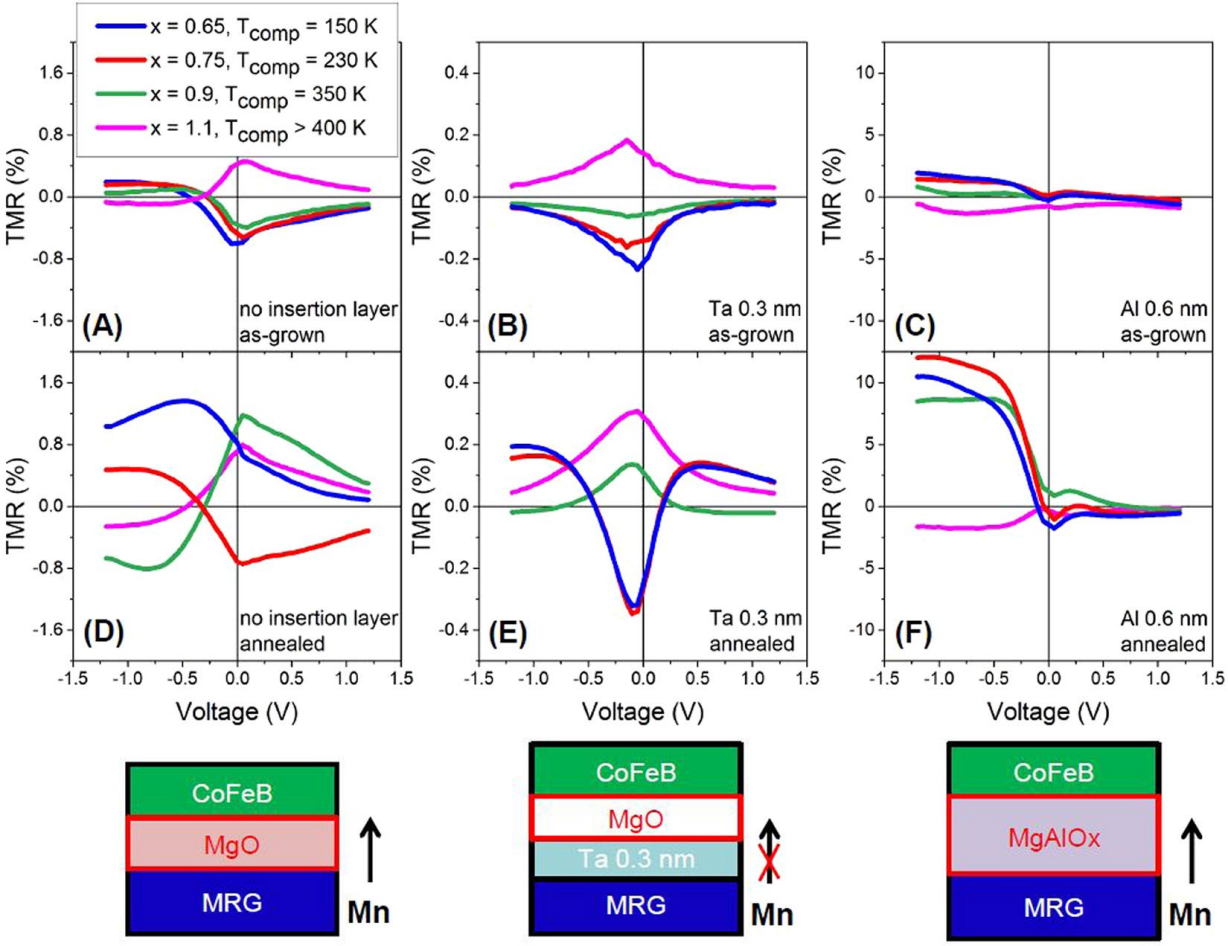
TMR as a function of bias voltage in MTJs with different MRG electrode composition. Panels (**A–C**) TMR (V) for as-grown samples; (**D**–**F**) for annealed during 1 hour at 325 C junctions. The inversion of the voltage dependence of TMR upon annealing is the shift of the compensation temperature of Mn_2_Ru_0.9_Ga from slightly below room temperature to slightly above.

Figure 5(A–F) shows the TMR ratio at room temperature as a function of applied bias. The asymmetry in TMR (V) is usually attributed to differences in the electronic states at the MRG/MgO and MgO/CFB interfaces^48–50^, and not to band structure effects. The samples with Ru concentration *x* = 1.1 (pink lines) showed an inverted bias voltage dependence compared to other compositions, confirming the model outlined above. This composition has a compensation point above room temperature (>400 K), inverting its TMR bias dependence. The samples with *x* = 0.9 (green lines) show a *T*_comp_ shift to room temperature with annealing for no diffusion barrier and the Ta barrier. With Al 0.6 nm, *T*_comp_ remains below room temperature for *x* = 0.9 even after annealing, this is due to the substantial reduction in *T*_comp_ with this diffusion barrier (Fig. 2(D)).

The shape of the TMR (V) response gives an insight into the interface of MRG and MgO. For multilayers with no diffusion barriers, the TMR response is low. In the case of MTJs without diffusion barrier (Fig. 5(A,D)) and with an Al 0.6 nm diffusion barrier (Fig. 5(C,F)), there is a clear asymmetry of the TMR response in bias voltage. For the Ta 0.3 nm diffusion barrier (Fig. 5(B,E)), the shape of the curve is similar to that observed in symmetric CoFeB/MgO/CoFeB tunnel junctions^17^. This is to be expected, if Ta is forming a stable metal interface with MRG. There can be no voltage applied to the MRG/Ta interface as the voltage would drop over the MgO/Ta interface. As a result, it is not possible to obtain an inverted TMR, as one can never apply the correct voltage to MRG yielding tunneling to the Mn_4*a*_ pocket. Regarding the magnitude of TMR, the highest TMR was detected for Al 0.6 nm insertion layer (12% after annealing). As this is an order of magnitude larger than for the case without diffusion barrier, this is attributed to the formation of a hybrid AlOx/MgO or a stable MgAl_2_O_4_ tunnel barrier^41,42^. No TMR was observed in the MTJs with Al 0.3 nm and Al 0.9 nm insertion layers. Since MgAl_2_O_4_ is a line compound that requires (almost) the exact stoichiometry of Mg, Al and O to form, then possibly, an Al 0.3 nm insertion is not enough and an Al 0.9 nm insertion is too much for the formation of a good quality MgAl_2_O_4_ tunnel barrier. The low TMR achieved in the samples without a diffusion barrier highlights that a diffusion barrier is needed. The Ta 0.3 nm diffusion barrier exhibits the lowest TMR response. As it is believed that Ta is forming a stable metal layer which prevents material diffusion, then the resultant TMR in a device with this diffusion barrier will be reduced significantly due to its short spin diffusion length (~1–3 nm)^51,52^. For samples with thicker Ta diffusion barriers, only the AMR response of CoFeB is observed, which further indicates that Ta is suppressing the spin polarization from the MRG reference layer. Although the TMR (V) behavior is consistent with the MRG density of states picture, resonant tunneling effects through the ultrathin insertion layer cannot be completely ruled out^53–55^.

The absence of a sign change of TMR, a thin MgO thickness on TEM, retention of *T*_comp_ and low RA product all clearly indicate that Ta 0.3 nm is a very effective diffusion barrier. Nevertheless, the TMR is still very low. Moreover, for samples with Mn_2_Ru_0.9_Ga bottom electrode annealed at 325 °C, the voltage dependence of the TMR also becomes inverted. Upon annealing, MRG loses some Mn, and the relative concentration of Ru increases, leading to an increase of *T*_comp_. This behavior can again be understood in terms of magnetotransport as being governed by one sublattice only. Shifting *T*_comp_ from above room temperature to below room temperature has the effect of inverting the sublattice moments with respect to the applied magnetic field, and as a result direction of *P*_eff_ and TMR as well.

In conclusion, ferrimagnetic half-metallic Mn_2_Ru_*x*_Ga integrated into MTJs demonstrates a low magnetic moment as well as, high coercivity (exceeding 5 T close to *T*_comp_). The largest TMR was reached for the samples with Al 0.6 nm layer inserted at the interface with MgO, possibly leading to the formation of a MgAl_2_O_4_ barrier. On the other hand, the insertion of this layer results in a considerable shift of *T*_comp_ towards lower temperatures. Insertion of a Ta 0.3 nm layer doesn’t change the composition of MRG but results in low TMR ratios due to a loss of spin polarisation within Ta. Ta is an ideal capping layer for MRG, but not an ideal insertion layer between MRG and MgO. Nevertheless, the results obtained using Ta spacers indicate that control of the MRG/barrier interface is possible, and if a spin-transparent insertion layer such as Hf^56^ is used, higher TMR should be achievable. Another possibility would be to use a conductive insertion layer such as TiN and rely on GMR effects to create modulation depth in future CPP STNOs. Although the choice of insertion layer heavily affects the Mn content in MRG and therefore leads to large changes in *T*_comp_, the half-metallic properties of MRG are retained in all devices, and the use of MRG as a free layer in STNOs with crossed anisotropy is still feasible. The effort for improved integration of Mn-based Heusler alloys in high-performance spintronic devices continues as this can open new technological and scientific domains.

## Methods

### Sample preparation

MRG-based MTJs were grown on MgO (001) single-crystal substrates by dc magnetron sputtering using a fully automated high-vacuum deposition system “Shamrock”, with a base pressure less than 1 × 10^−7^ mbar. MRG was grown by co-sputtering from a Mn_2_Ga and a Ru target. The growth temperature for the MRG electrode was in the range from 300 to 350 °C, the rest of the stack was deposited at room temperature. The samples were patterned into junctions, using standard UV lithography techniques and dry etching with secondary ion mass spectroscopy. Thermal evaporated SiO was used as interlayer insulating material. A Cr/Au top contact for electrical connections was defined by electron beam deposition and lift-off.

### Sample characterization

High-resolution TEM investigations were done with an image C_s_-corrected Titan 80–300 microscope (FEI) operated at an accelerating voltage of 300 kV. The element content of the film was checked during the dry etching with second ion mass spectrometer (SIMS). Magnetic properties of the unpatterned multilayer films were measured by quantum design superconducting quantum interference device (SQUID) with a maximum applied field of ±7T in the temperature range from 60 K to 400 K. All magneto-electrical measurements were carried out with a Keithley 2401 source-meter at room temperature.

## Data Availability

The datasets generated during and/or analyzed during the current study are available from the corresponding authors on reasonable request.
